# *In Vivo* quantitative imaging of angiogenesis-targeted PFOB nanoparticles in a hypercholesterol rabbit model using ^19^F-MRI with ultra-short echo time balanced SSFP

**DOI:** 10.1186/1532-429X-14-S1-M8

**Published:** 2012-02-01

**Authors:** Matthew J Goette, Anne H Schmieder, Todd A Williams, John S Allen, Jochen Keupp, Gregory Lanza, Samuel A Wickline, Shelton D Caruthers

**Affiliations:** 1 School of Medicine, Washington University in St. Louis, St. Louis, MO, USA; 2 Philips Research Europe, Hamburg, Germany

## Summary

Herein, initial results are presented as obtained in a hypercholesterol rabbit model with the simultaneous ^19^F/^1^H balanced UTE-SSFP technique and using α_ν_β_3_-targeted PFOB nanoparticles to establish the feasibility of high sensitivity MR molecular imaging of Gd-free, fluorine-based, clinically-relevant contrast agents.

## Background

α_ν_β_3_-integrin targeted nanoparticle (NP) emulsions have been shown to detect and quantify angiogenesis and anti-angiogenic therapy in small animal models of atherosclerosis. While these NP were visualized in high resolution pre- and post-injection ^1^H-MRI via a Gadolinium (Gd) chelate, we seek to image the perfluoro-octyl bromide (PFOB) core directly via ^19^F MR. Early *in vivo* successes of ^19^F MR molecular imaging exploited the single resonance peak of perfluoro-crown-ether. However, PFOB, which is the more clinically-relevant NP with a better-understood human safety profile, has a more complex spectrum with seven ^19^F resonance peaks and multiple relaxation conditions, leading to chemical shift artifact and intra-voxel destructive interference. We hypothesize that a new technique—simultaneous dual-frequency ^19^F/^1^H ultra-short echo time (UTE) balanced steady state free precession (b-SSFP) sequence with 3D radial readout—will allow efficient, sensitive imaging of the complex PFOB signal without the need for Gd and in sufficient resolution to discern the anatomy even in the presence of cardiac and respiratory motion.

## Methods

The study was performed using a dual-tuned transmit/receive surface coil (7×12cm) on a 3T clinical whole-body scanner (Achieva, Philips Healthcare) modified for truly-simultaneous ^19^F/^1^H operation. Male New Zealand White rabbits were fed high cholesterol chow for 20 weeks. Imaging was performed 2h post-injection of 1.0ml/kg of the α_ν_β_3_-targeted PFOB-NP. A UTE b-SSFP sequence with simultaneous ^19^F/^1^H excitation and 3D radial readout was acquired at six time points post-injection with the following parameters: FOV=140mm, matrix 112^3^, isotropic voxel Δx=1.25mm, α=30°, excitation bandwidth exBW=9kHz, pixel bandwidth pBW=900Hz, TR=2.0ms, TE=100μs (FID sampling), total scan time 28 min. The radial k-space data was reconstructed at full resolution for the ^1^H component, and at lower resolutions with higher signal to noise for the ^19^F component (Nyquist radius 7%). ^19^F-data from subsequent time points were combined to provide an image of the spatial NP distribution. The ^19^F-signal was calibrated for ^19^F concentrations using an agar phantom containing PFOB-NP at 150mM_19F_.

## Results

**Figure 1:**
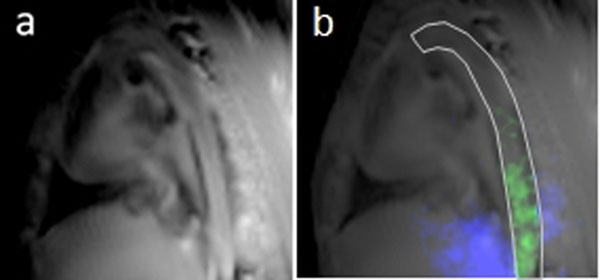
Simultaneous ^19^F/^1^H molecular imaging of angiogenesis targeted perfluoro-octyl-bromide nanoparticles in a rabbit model of atherosclerosis using 3D radial balanced UTE-SSFP. Proton images (a) with 1.25mm isotropic voxels show anatomy, upon which ^19^F image can be over-laid (b). The ROI in (b) is surrounding the aorta, which has a diameter of about 5mm. The ^19^F overlay within the aortic region is in green, and extra-aortic ^19^F signal is blue.

*In vivo* imaging of angiogenesis-targeted PFOB nanoparticles was successful using the ^19^F/^1^H UTE b-SSFP sequence. Figure 1a shows an example of the proton image quality in a selected slice at the aorta, which is robust against motion due to the simultaneous 3D radial acquisition. The isotropic voxel allows multi-planar reformatting for visualizing anatomy and prescribing ROIs for analyzing the directly-corresponding ^19^F NP signal. In this example, α_ν_β_3_-targeted PFOB-NP were detected in the aorta ROI (Fig.1b) in concentrations ranging from 10 to 16mM.

## Conclusions

Dual frequency ^19^F/^1^H radial 3D balanced ultra-short TE is a versatile pulse sequence that allows high-sensitivity, high-resolution *in vivo* detection of angiogenesis-targeted PFOB-NP despite the possible complex resonant peak interaction.

## Funding

NIH R01 HL073646.

